# A compact very wideband amplifying filter based on RTD loaded composite right/left-handed transmission lines

**DOI:** 10.1186/s40064-015-1529-y

**Published:** 2015-11-25

**Authors:** Mahmoud O. Mahmoud Abu-marasa, Hala Jarallah El-Khozondar

**Affiliations:** Electrical Engineering Department, Islamic University of Gaza, Gaza, Palestine

**Keywords:** CRLH-TL, Left-handed materials, RTD, Filter, Amplifier, SRRs

## Abstract

The composite right/left-handed (CRLH) transmission line (TL) is presented as a general TL possessing both left-handed (LH) and right-handed (RH) natures. RH materials have both positive permittivity and positive permeability, and LH materials have both negative permittivity and negative permeability. This paper aims to design and analyze nonlinear CRLH-TL transmission line loaded with resonant tunneling diode (RTD). The main application of this design is a very wideband and compact filter that amplifies the travelling signal. We used OrCAD and ADS software to analyze the proposed circuit. CRLH-TL consists of a microstrip line which is loaded with complementary split-rings resonators (CSRRs), series gaps, and shunt inductor connected parallel to the RTD. The designed structure possess a wide band that ranges from 5 to 10.5 GHz and amplifies signal up to 50 %. The proposed design is of interest to microwave compact component designers.

## Background

The resonant tunneling diodes (RTD) that pocesses negative differential resistance region have attracted a lot of attention. RTD has high switching speed up to 2.2 THz (Ling 2014). It has been shown that it is possible to monolithically integrate RTD with an optical waveguide electroabsorption modulator (Figueiredo et al. 2001), with laser diode (Slight et al. 2008) and with transmission line analog to digital converter (El-Khozondar et al. 2013a) or signal reshaping (El-Khozondar et al. 2013b).

In 2004, Falcone et al. (2004a) synthesized the first MTMs from CSRRs. Later, the first LH line based on CSRRs was achieved by Falcone et al. (2004b). In a study performed in 2007 (Gil et al. 2007), researchers used LH/RH transmission lines based on CSRRs to achieve a compact very wideband filter ranges between 5-10 GHz. The TL consists of three units.

In a recent work by Maezawa et al. (2010), an amplified signal is achieved when a signal travels through CRLH transmission line loaded with RTD pairs. Authors achieved wide frequency range between 100 and 200 GHz. Their TL consists of 160 units.

The main goal of this paper is to study left- and right-handed transmission line based on CSRRs loaded with RTD to obtain ultra-wideband (UWB) and filter with amplified output signal. Our model is a modification to the hybrid model (Gil et al. 2007) by loading TL with RTD. The hybrid model is a CRLH-TL composed of a microstrip line loaded with complementary split-rings resonators (CSRRs), series gaps, and shunt inductor (Gil et al. 2007). In our work, we are looking to obtain a compact ultra-wideband filter that modifies the travelling signal. In Section II, the I–V characteristic of the RTD is presented. A prototype of the device is introduced in section III. Conclusion is given in section IV.

## Resonant tunneling diode

**Fig. 1:**
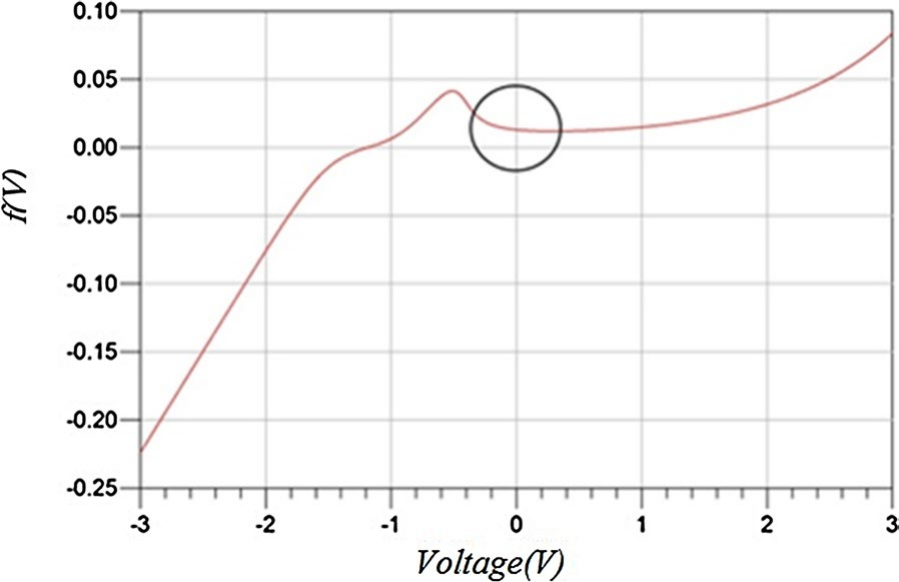
I–V characteristics of RTD. The *circle marks* the negative differential resistance (NDR) region

Resonant tunneling diode (RTD) has unique applications due to the high switching speed and its negative differential resistance. Figure 1 shows RTD I-V characteristic curve.

The characteristic RTD curve presented in Fig. 1 show the nonlinear behavior of the RTD. There are three regions: the circled one is the region with negative resistance while the regions below and above the negative resistance region has positive resistance value. The characteristic curve shown in Fig. 1 is formulated by the following expression (Reddy 1997).

1 $$f\left( V \right) = A\ln \left( \alpha \right) + H\left( {e^{{n_{2} qV\left( t \right)/K_{B} T}} - 1 } \right)$$

where $\alpha = \left[ {\frac{{1 + e^{{q\left( {B - C + n_{1} {\kern 1pt} V\left( t \right)} \right)/K_{B} T}} }}{{1 + e^{{q\left( {B - C - n_{1} {\kern 1pt} V\left( t \right)} \right)/K_{B} T}} }}} \right] \left[ {\frac{\pi }{2} + \tan^{ - 1} \left( {\frac{{C - n_{1} {\kern 1pt} V(t)}}{D}} \right)} \right]$, *f*(*V*) is the current density as function of *V* which is the voltage cross RTD, *K*_*B*_, *T*, and *q* are Boltzmann constant, temperature in Kelvin and electron charge in coulombs, respectively. *A*, *B*, *C* and *D* are the dependent parameters on the device’s physics. The experimental RTD I–V characteristic is fitted using the physics based description of the RTD equation in Fig. 1. The fitting parameters are taken from (Slight et al. 2008) as follows, *A* = 6.48 × 10^−3^, *B* = 0.0875, *C* = 0.1449, *D* = 0.02132, *H* = 7.901x10^−4^, *n*_*1*_ = 0.1902, *n*_*2*_ = 0.0284, and *T* = 300 *K*.

## The proposed filter-amplifier

**Fig. 2:**
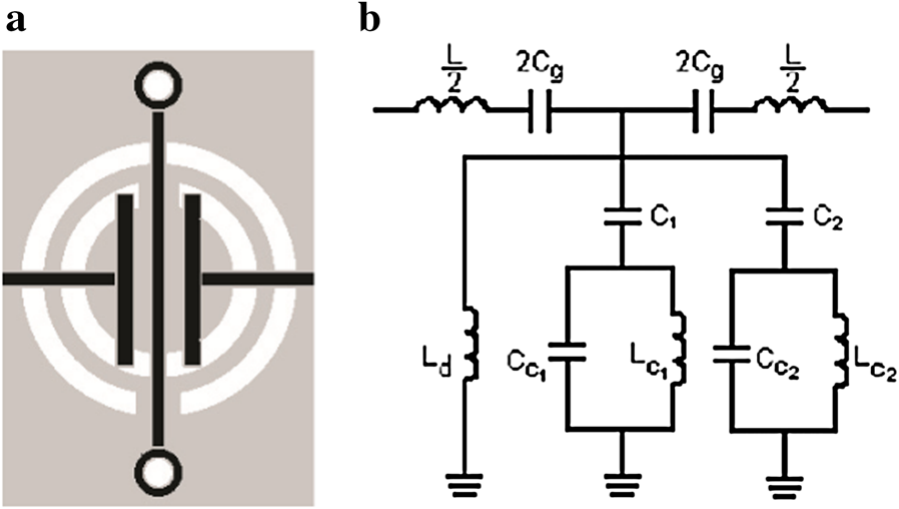
**a** The structure of the hybrid left-handed cell. **b** Equivalent circuit of one hybrid left-handed cells

The proposed filter-amplifier is constructed by combining the RTD with CRLH-TL based on hybrid CSRR (Gil et al. 2007). The filter introduced in (Gil et al. 2007) consists of four cells with active area below 1 cm^2^, bandwidth that fluctuates from 5 to 10 GHz and ripple below 1 dB. Figure 2 presents the unit cell and its equivalent lumped circuit. The unit cell is designed with the series resonance frequency as close as possible to the higher resonance of the shunt impedance (ω_S_ = ω_PH_).

**Fig. 3:**
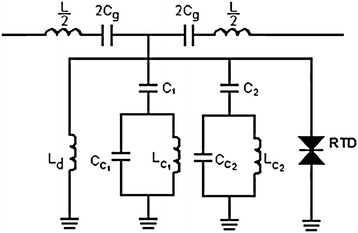
Equivalent circuit model of the unit cell (Fig. 2) loaded with RTD

**Fig. 4:**
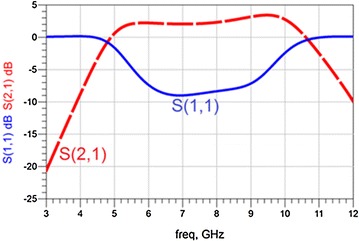
Simulated reflection and transmission coefficients of a single cell loaded with RTD

We design our filter-amplifier by adding RTD parallel to L_d_ as illustrated in Fig. 3. The parameters are (Gil et al. 2007): L = 2.6 nH, Cg = 0.2pF, L_d_ = 5.4 nH, C_1_ = 305 pF, C_C1_ = 0.65pF, L_C1_ = 0.55nH, C_2_ = 0.21pF, C_C2_ = 0.32 pF, and L_C2_ = 0.27 nH. The RTD was biased in the NDR region using a series variable DC voltage supply. Figure 4 demonstrates the simulated reflection and transmission coefficient of a single cell with RTD, corresponding to the filter (Fig. 3) using Agilent ADS program. Moreover, it can be seen from Fig. 4 that the effect of RTD on transmission coefficient (S_21_) goes above 0 dB. This means that the output power of the filter is bigger than the input power.

The proposed filter allows a signal to pass in wide range starting at 5 GHz and ends at 10.5 GHz. In addition, this filter is capable to amplify the signal.

**Fig. 5:**
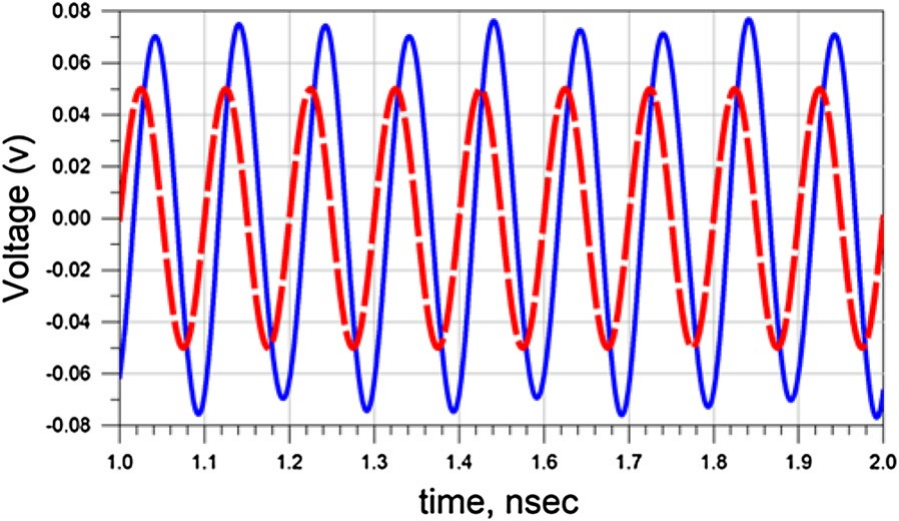
Simulation result to the equivalent circuit shown in Fig. 3

We simulated the value of the output signal and compared it with the value of the input signal to understand the amplification behavior of our filter. Figure 5 presents the transient response of the input and output signals of the designed filter (Fig. 3) at 9 GHz when DC biasing is set to be 1.18 V. The dashed line refers to the input signal and the sold line refers to the output signal of the filter. The amplification of the input signal is clearly observed. The peak value of the input value is 50 mV while the amplified output signal has a peak value equal to 75 mV.

We have noticed that the amplification is 50 %. Further, we realized that we could not excite our filter with input more than 300 mV, because above this voltage the filter changes the shape of the input signal.

To understand the effect of RTD, we have focused on the negative resistance region (RD). From Fig. 1, we noticed that RD region ranges from −0.1 to 0.1 V. Additionally, the relationship between voltage and currant in the RD region is almost linear. As a result, we can replace the original equation of RTD by approximated Eq. (2):

2 $$I = \left( {slope} \right)V = A^{\prime} V$$

where *A*′ is the slop of currant at the working regain and *V* is the voltage drop at RTD terminal. The slop is approximately equal to 0.09. To examine the approximated equation of RTD, we have replaced the RTD in Fig. 3 by a variable current source which depends on the voltage across the terminals of RTD such as I = 0.09 *V*.

**Fig. 6:**
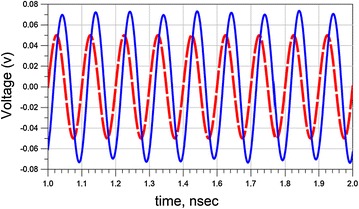
Reports the simulation result of the equivalent circuit with approximated RTD (Eq. 2) at 9 GHz. The *dash line* refers to the input signal and the *sold line* refers to the output signal of the filter

Figure 6, displays the simulated results when RTD is replaced with current source. The result is similar to the results shown in Fig. 5. Meaning that the output signal is amplified compared to the input signal.

Thus, we conclude that for our calculations, the simplified model [Eq. (2)] of RTD can replace the complex representation of RTD characteristic that appears in Eq. (1).

## Conclusion

We have designed a compact UWB filter-amplifier by adding RTD to CRLH-TL hybrid unit cell. The bandwidth ranges from 5 to 10.5 GHz and the output signal is amplified compared to the input signal. This is a unique presentation of a filter-amplify based on CRLH-TL loaded with RTD. The designed filter is useful to get rid of the interfering signals below the frequency region of interest. It also can amplify the signal at voltages below 0.3 V. Thus, this filter is good in antenna applications.
